# Protective effect of *Limosilactobacillus fermentum* HFY06 on dextran sulfate sodium-induced colitis in mice

**DOI:** 10.3389/fmicb.2022.935792

**Published:** 2022-09-12

**Authors:** Bihui Liu, Lei Yang, Ya Wu, Xin Zhao

**Affiliations:** ^1^Collaborative Innovation Center for Child Nutrition and Health Development, Chongqing University of Education, Chongqing, China; ^2^Chongqing Engineering Research Center of Functional Food, Chongqing University of Education, Chongqing, China; ^3^Chongqing Engineering Laboratory for Research and Development of Functional Food, Chongqing University of Education, Chongqing, China; ^4^College of Biological and Chemical Engineering, Chongqing University of Education, Chongqing, China; ^5^Department of Urology, First Affiliated Hospital of Chengdu Medical College, Chengdu, China

**Keywords:** *Lactobacillus fermentum*, dextran sulfate sodium (DSS), colitis, inflammation, probiotics

## Abstract

Ulcerative colitis is one of the main gastrointestinal diseases that threaten human health. This study investigated the effect of *Limosilactobacillus fermentum* HFY06 (LF-HFY06) on dextran sulfate sodium (DSS)-induced murine colitis. The protective effect of LF-HFY06 was evaluated by examining the length and histopathological sections of colon, related biochemical indicators, and genes related to inflammation. Direct and microscopic observations showed that LF-HFY06 increased the length of the colon and ameliorated the pathological damage induced by DSS. The biochemical indicators showed that LF-HFY06 enhanced the activities of antioxidant enzymes total superoxide dismutase (T-SOD) and catalase (CAT) in serum, while reducing the level of malondialdehyde (MDA). It was also observed that the serum inflammatory cytokines levels of tumor necrosis factor-α (TNF-α), interferon (IFN)-γ, interleukin (IL)-1β, IL-6, and IL-12 were decreased, and the anti-inflammatory cytokine IL-10 level was increased. The qPCR experiment revealed that LF-HFY06 downregulated the mRNA expression levels of nuclear factor-κB-p65 (*Rela*), *Tnf*, *Il 1b*, *Il 6*, and prostaglandin-endoperoxide synthase 2 (*Ptgs2*) in colon tissues, and upregulated the mRNA expression of NF-κB inhibitor-α (*Nfkbia*) and *Il 10*. These data indicated that LF-HFY06 inhibited inflammation through the NF-κB signaling pathway to prevent the occurrence and development of colitis. This research demonstrates that probiotics LF-HFY06 have the potential to prevent and treat colitis.

## Introduction

Ulcerative colitis is a non-specific inflammatory disease primarily involving the rectum, colonic mucosa, and submucosa (Feuerstein et al., 2019). The specific cause of ulcerative colitis is not completely clear at present, and the main factors affecting its pathogenesis are genetic and environmental factors, immunity, and gut microbes (Kaplan, 2015; Liu and Stappenbeck, 2016). Although the incidence of inflammatory bowel disease (IBD) in North America and Europe has stabilized in recent years, its incidence has been rising in the newly industrialized countries of Africa, Asia, and South America (Seyedian et al., 2019; Mak et al., 2020). The first-line drugs currently used in clinical are mainly 5-aminosalicylic acids, glucocorticoids, biological agents, and immunosuppression. However, the above-mentioned drugs have the problems of easy recurrence after drug withdrawal or toxic and side effects caused by long-term use (Damião et al., 2019; Cohen and Weisshof, 2020). Thus, it is very important to study new therapeutical alternatives for the prevention and treatment of IBD. In this context, probiotic microorganisms have been explored as an alternative therapeutic approach against intestinal inflammation (Jang et al., 2019; Hrdı et al., 2020; Barroso et al., 2022).

Probiotics as living microorganisms provide a health benefit to the host by regulating the intestinal microbiota (Juarez et al., 2013), inhibiting the colonization of pathogenic bacteria, regulating immunity, and secreting antibacterial substances (Kostic et al., 2014; Sonnenburg and Bäckhed, 2016; Bron et al., 2017; Shen et al., 2018). The most common probiotics are *Limosilactobacillus* and *Bifidobacterium* (Lee et al., 2018; Robertson et al., 2020; Yadav et al., 2022). A lot of research shows that the probiotics that can be used for the prevention and treatment of colitis include *Lactiplantibacillus plantarum* (Liu et al., 2011), *Lacticaseibacillus rhamnosus* (Zocco et al., 2006), *Lactobacillus bulgaricus* (Takamura et al., 2011), and *Bifidobacterium* (Nishida et al., 2018). They prevent or relieve colitis through antioxidant, immunomodulatory, and change the composition of gut microbiota. Therefore, the rational application of probiotics is an important strategy to prevent and treat colitis.

*Limosilactobacillus fermentum* HFY06 (LF-HFY06) is a potential probiotic strain isolated from yak yogurt, which was collected from the Aba Tibetan and Qiang Autonomous Prefecture of Sichuan province. In previous studies, LF-HFY06 has the effect of alleviating CCl_4_ (carbon tetrachloride)-induced liver injury and d-galactose-induced oxidative stress and inflammation in mice (Li et al., 2020, 2021a,2021b). The mechanism of LF-HFY06 may be involved in the upregulation of antioxidant genes (*Nrf2*, *Gclc*, *Sod1*, *Sod2*, and *Cat*) expression and downregulation of inflammation-related genes (*Rela*, *Tnf*, and *Ptgs2*) expression. In a previous study, we reported the effect of the synbiotic composed of arabinoxylan and HFY06 on colitis. However, no comparative study with clinically positive drugs has been performed to further explain its effect. Based on the increased incidence of colitis in the above-mentioned regions, we selected the LF-HFY06 strain that has shown certain physiological activity to further explore its role in colitis.

## Materials and methods

### Source of strain

LF-HFY06 was isolated from Hongyuan yak yogurt, a unique product of Aba Tibetan and Qiang Autonomous Prefecture in Sichuan, China. NCBI’s Basic Local Alignment Search Tool (BLAST) was used to identify the experimental strain LF-HFY06, which has been preserved in the China General Microbiological Culture Collection Center (CGMCC, Beijing, China; CGMCC No. 16636).

### Bacteria growth conditions

The LF-HFY06 (100 μL) was grown in 5 mL of MRS broth at 37°C for 24 h. To prepare the bacteria doses, the optical density of the bacterial suspension was measured at 595 nm, and its concentration was adjusted to 1 × 10^9^ CFU/mL with normal saline.

### Animals and experimental procedure

**FIGURE 1:**
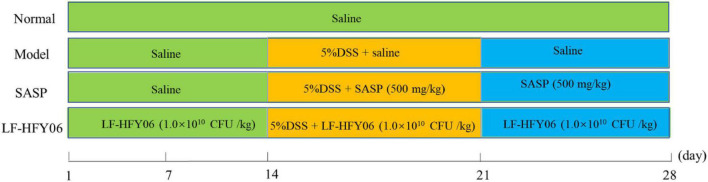
Animal experiment design of this study.

Animal experiments were approved by the Ethics Committee of Chongqing Collaborative Innovation Center for Functional Food (IACUC Number: 201906002B). All mice were housed at a room temperature of 20–22°C, 50 ± 10% humidity, under a 12 h diurnal light/dark cycle. During the experiment, the mice were allowed to eat standard rat chow and drink water freely. After a week of adjustable feeding, the experiment was performed as shown in Figure 1. Forty male Kunming mice (Chongqing Medical University, 6 weeks old) were randomly divided into four groups, namely, a normal group, model group, LF-HFY06 group (LF-HFY06), and salicylazosulfapyridine (SASP) group (Chen et al., 2017). The mice in the LF-HFY06 group received intragastric administration of 1.0 × 10^9^ CFU/mL of LF-HFY06 (0.1 mL/10 g) every day for 28 days. From days 1 to 14, the mice in the LF-HFY06 group were treated as described above, and the other mice were treated with saline solution (0.1 mL/10 g) by intragastric administration. From days15 to 21, the mice in the normal group received sterile distilled water every day, and the other mice were given 5% DSS (molecular mass, 36–50 kDa; MP Biomedicals, Santa Ana, CA, United States) solution instead of sterile distilled water. At the same time, the mice in the SASP group received intragastric administration of 50 mg/mL (0.1 mL/10 g) SASP (Shandong Huimeng Biotech Co., Ltd., Shandong, China). From days 22 to 28, the mice in the normal group and model group received sterile distilled water, and the mice in LF-HFY06 and SASP groups received intragastric administration as described above. On the day 29, the mice were anesthetized with ether for blood sampling. Afterward, the mice were sacrificed and the colon section was collected. The blood was centrifuged (4,000 rpm for 10 min at 4°C) to obtain the serum. The colon and serum were kept in an ultra-low temperature refrigerator (−80°C) for further testing.

### Assessment of disease activity index score of colitis

The body weight loss, stool consistency, and rectal bleeding were observed during the modeling period (15–21 days). Each indicator is scored according to Table 1 and the disease activity index (DAI) of mice was calculated (Yao et al., 2010). DAI = (combined score of weight loss, stool consistency, and bleeding)/3.

**TABLE 1:** Evaluation of disease activity index (DAI) scores.

Score	Weight loss	Stool consistency	Occult/gross bleeding
0	(-)	Normal	Normal
1	1–5%		
2	6–10%	Loose	Occult bleeding
3	11–15%		
4	>15%	Diarrhea	Gross bleeding

### Histological analysis

The portion of the colon tissues was processed by the following procedure: 10% formalin solution fixation, dehydration, paraffin embedding, sectioning, hematoxylin, and eosin staining. Finally, the colon sections were fixed in neutral gum, and the pathological changes were examined by optical microscope (BX43 microscope, Olympus, Tokyo, Japan). Pathological damage of colon tissue was scored based on specific criteria shown in Table 2 (Takagi et al., 2011). The total histological score was the sum of the epithelium and infiltration scores (total score = E + I), and thus ranged from 0 to 8.

**TABLE 2:** Evaluation of histological scores.

Epithelium (E)	Score	Infiltration (I)	Score
Normal morphology	0	No infiltration	0
Loss of goblet cells	1	Infiltration around crypt bases	1
Loss of goblet cells in large areas	2	Infiltration reaching the muscularis mucosa	2
Loss of crypt	3	Extensive infiltration reaching the muscularis mucosa and thickening of the mucosa with abundant edema	3
Loss of crypts in large areas	4	Infiltration of the submucosa	4

### Total-superoxide dismutase, catalase, and malondialdehyde levels in the serum of mice

The level of antioxidants markers T-SOD, CAT, and MDA in serum was performed using conventional biochemical kits (Nanjing Jiancheng Bioengineering Institute, Nanjing, China), according to the recommended instructions.

### Cytokines levels

The determination of the serum cytokine levels TNF-α, IFN-γ, IL-1β, IL-6, IL-12, and IL-10 was processed under the instructions of the ELISA kit (Beijing Chenglin Biotechnology Co., Ltd., Beijing, China).

### Colonic gene expression analysis

The distal colonic (100 mg) was grounded in 1 mL of TRIzol reagent (Invitrogen, Carlsbad, CA, United States) to afford tissue homogenate, and then it was added with 200 μL chloroform. After the mixed solution was centrifuged at 14,000 rpm/min at 4°C for 15 min, the residue was discarded and isopropyl alcohol (500 μL) was subsequently added to the supernatant. The solution was fully mixed and placed at 4°C for 15 min, followed by centrifuging at 14,000 rpm/min and 4°C for 20 min. After discarding the supernatant, the precipitate was washed with 75% ethanol solution. After centrifugation once more, the upper water phase was removed, and the RNA precipitate was dissolved in 20 μL of enzyme-free water. The concentration and purity of the total RNA were tested *via* ultra-microspectrophotometry (Nano-100, All for Life Science, Hangzhou, Zhejiang, China).

To prepare a cDNA template, 1 μg/μL of RNA solution and a reverse transcription kit (Tiangen Biotech Co., Ltd., Beijing, China) were employed according to the instructions. A solution of cDNA (1 μg/μL, 1 μL) and SYBR Green PCR Master Mix (10 μL) was added with upstream and downstream primers (1 μL, Table 3) to afford a qPCR reaction solution (Zhou et al., 2019). Then, amplification was performed using the Applied Biosystems StepOnePlus Real-Time PCR Instrument (Thermo Fisher Scientific Co., Ltd., MA, United States). The cycling parameters were: 95°C for 90 s, 40 cycles of 95°C for 30 s, 60°C for 30 s, 72°C for 30 s, then, 95°C for 30 s, and 55°C for 35 s. With β-actin as a housekeeping gene, the 2^–Δ^ ^Δ^ *^Ct^* method was selected to calculate the relative expression of the related gene (Livak and Schmittgen, 2001).

**TABLE 3:** Sequences of primers used in this study.

name	Sequence	bp	Genbank accession no.
*Rela*	Forward: 5′-ATGGCAGACGATGATCCCTAC-3′	167	XM_006501107
	Reverse: 5′-CGGAATCGAAATCCCCTCTGTT-3′		
*Nfkbia*	Forward: 5′-TGAAGGACGAGGAGTACGAGC-3′	127	XM_003987542
	Reverse: 5′-TGCAGGAACGAGTCTCCGT-3′		
*Tnf*	Forward: 5′-CTGAACTTCGGGGTGATCGG -3′	122	XM_021149735
	Reverse: 5′-GGCTTGTCACTCGAATTTTGAGA-3′		
*Il 1b*	Forward: 5′-GAAATGCCACCTTTTGACAGTG -3′	116	NM_008361
	Reverse: 5′-TGGATGCTCTCATCAGGACAG-3′		
*Il 6*	Forward: 5′-CTGCAAGAGACTTCCATCCAG-3′	131	XM_021163844
	Reverse: 5′-AGTGGTATAGACAGGTCTGTTGG-3′		
*Il 10*	Forward: 5′-CTTACTGACTGGCATGAGGATCA-3′	101	XM_021175612
	Reverse: 5′-GCAGCTCTAGGAGCATGTGG-3′		
*Ptgs2*	Forward: 5′-GGTGCCTGGTCTGATGATG-3′	116	MW395257
	Reverse: 5′-TGCTGGTTTGGAATAGTTGCT-3′		
*Actb*	Forward: 5′-AACTCCATCATGAAGTGTGA-3′	247	XM_049111166
	Reverse: 5′-ACTCCTGCTTGCTGATCGAC-3′		

Rela, nuclear factor-κB p65; Nfkbia, NF-κB inhibitor α; Tnf, tumor necrosis factor-α; Il 1b, interleukin-1β; Il 6, interleukin-6; Il 10, interleukin-10; Ptgs2, prostaglandin-endoperoxide synthase 2; Actb, beta-actin.

### Statistical analysis

The experimental data analysis was accomplished in SPSS 17.0 (SPSS Inc., Chicago, IL, United States) and GraphPad Prism 7 statistical software (Graph Pad Software Inc., La Jolla, CA, United States). The mean ± standard deviation was the form to express the results. Comparisons among groups were obtained by one-way analysis of variance (ANOVA) followed by Tukey’s test, in which *P* < 0.05 indicated a significant difference.

## Results

### Colon length

**FIGURE 2:**
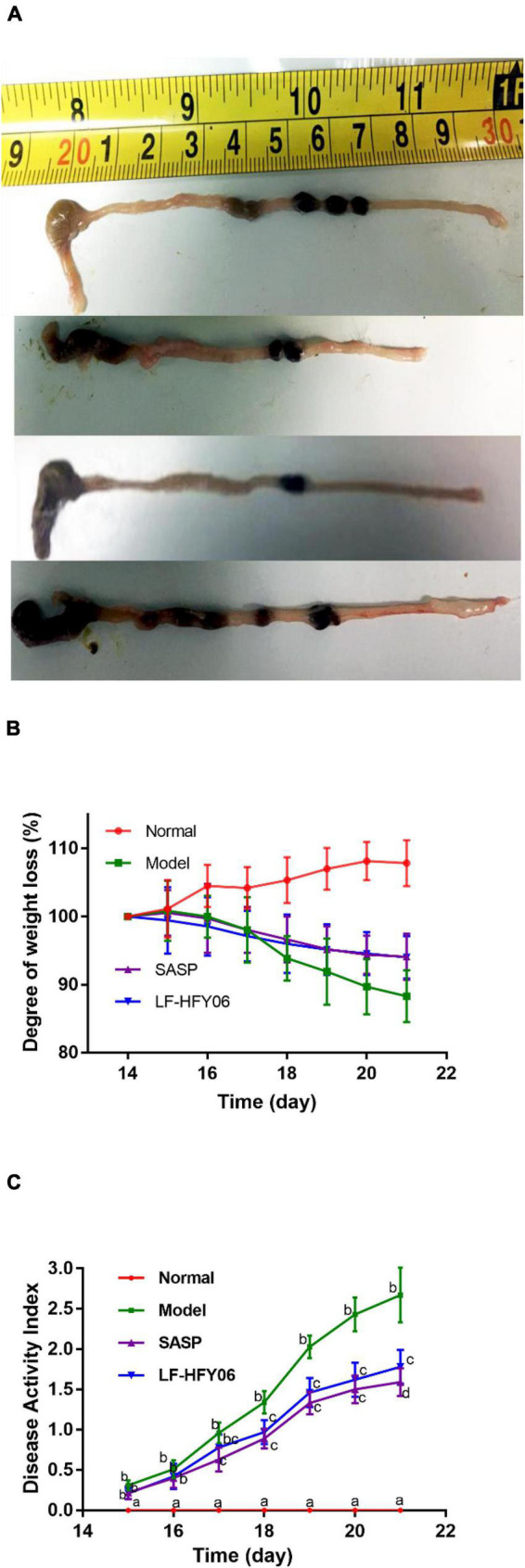
**(A)** The general appearance of colon tissue. **(B)** The degree of weight loss, ^a–*c*^Mean values with different letters on the same day indicate a significant difference after Tukey’s test (*P* < 0.05). **(C)** Disease activity index of mice in different groups. ^a–*d*^Mean values with different letters on the same day indicate a significant difference (*P* < 0.05) after two-way ANOVA.

Figure 2A shows the general appearance of colon tissue after the LF-HFY06 treatment. The colon length of mice in the model group was 7.86 ± 0.64 cm, which was significantly shorter compared with the normal group (9.82 ± 0.52 cm) (*P* < 0.05). Treatment groups significantly increased the colon length (LF-HFY06: 9.3 ± 0.64 cm; SASP: 9.28 ± 0.71 cm) when compared to the model mice group (*P* < 0.05).

### Assessment of disease activity index

During the experimental period, a gradual weight increase was observed for the animals in the normal group, while a significant weight loss was observed for the model group. Treatment with LF-HFY06 and SASP was able to attenuate this weight loss (Figure 2B). DAI scores of mice in each group are shown in Figure 2C. There was no significant difference between the treatment groups and model group on days 15–16. On day 17, DAI increased significantly in the model group (Figure 2C, *P* < 0.05), and the increasing trend was faster than that in the treatment groups. However, no significant difference was observed in DAI between the treatment groups during the establishment of the colitis model (days17–20; *P* > 0.05).

### Histological analysis

**FIGURE 3:**
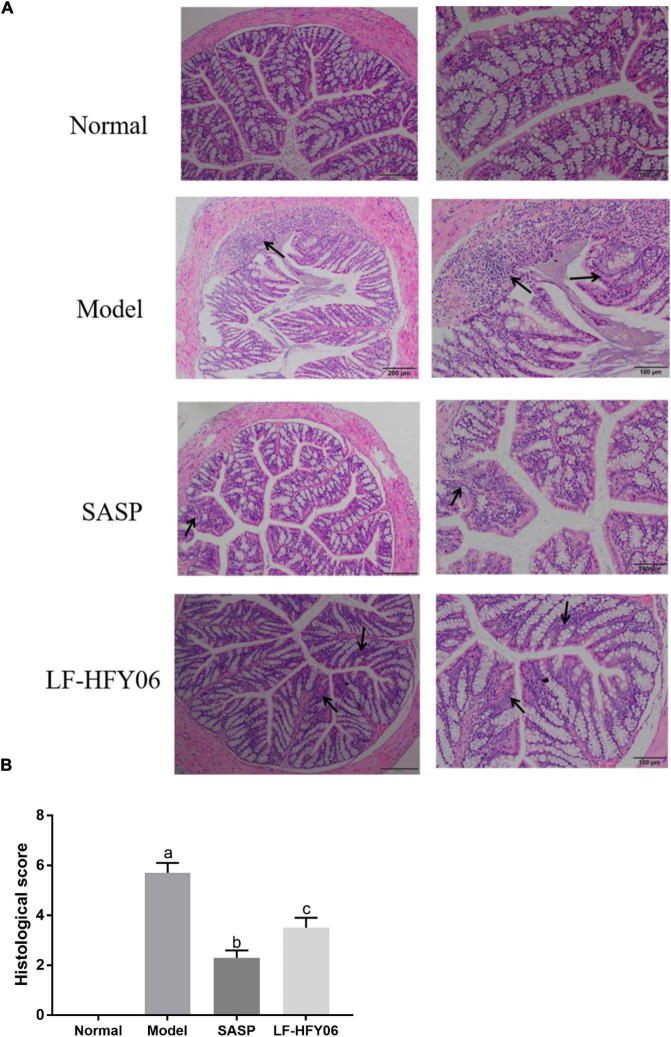
**(A)** Histopathological examination of colon tissue sections. **(B)** Histological disease score of colon tissue sections in mice of the different groups.

As shown in Figure 3, the colonic mucosal epithelial cells in the healthy mice were intact, and the glands were arranged in an orderly manner. Also, it was obvious that the crypts were normal with no ulcers. However, the mice in the model group exhibited severe colonic mucosal erosion, and the glands were disorderly arranged. Many crypts were dramatically destroyed and goblet cells were drastically reduced, accompanied by inflammatory cell infiltration (Figure 3A, black arrow). After the administration of LF-HFY06 and SASP, only a few ulcers appeared, and the crypts and the goblet cells were relatively complete with neatly arranged glands (Figure 3A, black arrow). The injury in the treatment group was less severe than that of the model group. Compared to the model group, the histological score of the treatment group was significantly lower (*P* < 0.05). The SASP group had a strikingly lower histological score than the LF-HFY06 group (*P* < 0.05; Figure 3B).

### Total-superoxide dismutase, catalase, and malondialdehyde levels in the serum of mice

The activities of T-SOD and CAT antioxidant enzymes in the model mice receiving DSS were significantly decreased, while the MDA production was significantly increased compared to those in the healthy mice (*P* < 0.05; Table 4). In contrast to the model group, LF-HFY06 and SASP significantly increased the activities of SOD and CAT, and significantly reduced the amount of MDA (*P* < 0.05; Table 4).

**TABLE 4:** Serum levels of T-SOD, CAT, and MDA in mice of each group.

Group	Normal	Model	SASP	LF-HFY06
T-SOD (U/mL)	218 ± 11^a^	127 ± 11^b^	184 ± 19^c^	198 ± 14^c^
CAT (U/mL)	57.6 ± 9.4^a^	17.9 ± 3.6^b^	43.2 ± 6.2^c^	35.2 ± 3.9^c^
MDA (nmol/mL)	3.8 ± 1.0^a^	13.1 ± 1.9^b^	6.9 ± 0.5^c^	6.8 ± 1.1^c^

Values are presented as the mean ± standard deviation (n = 8/group). Normal, mice were treated with saline; Model, mice were treated with 5% DSS; SASP, mice were treated with 50 mg/mL of salicylazosulfapyridine + 5% DSS; LF-HFY06, mice were treated with 1.0 × 10^9^ CFU/mL of LF-HFY06 + 5% DSS; *^a–c^*significant difference (P < 0.05) was indicated by different letters in the same row after Tukey’s test.

### Serum TNF-α, IL-1β, IL-6, IL-12, IFN-γ, and IL-10 levels in mice

A healthy intestinal barrier is critical for intestinal health, of which mucosal epithelial cells are an important part. When intestinal inflammation occurs, intestinal mucosal epithelial cells exert innate immune function, and thus, IL-4, IL-6, IL-10, and other cytokines in the tissue participate in the immune regulation response (Stevens et al., 1992). The pro-inflammatory cytokines TNF-α, IL-1β, IL-6, IL-12, and IFN-γ levels in mice with DSS-induced colitis were apparently higher than those of mice in the normal group, but anti-inflammatory cytokine IL-10 was lower (Table 5, *P* < 0.05). In comparison with the DSS model group, the pro-inflammatory cytokines TNF-α, IL-1β, IL-6, IL-12, and IFN-γ in the treatment groups showed a decreasing trend, and the anti-inflammatory factor IL-10 showed an increasing trend (Table 5, *P* < 0.05). The results showed that the inhibition of inflammation of LF-HFY06 was comparable to that obtained with SASP.

**TABLE 5:** Serum levels of TNF-α.

Group	Normal	Model	SASP	LF-HFY06
TNF-α (ng/L)	993 ± 82^a^	1,415 ± 45^b^	1,168 ± 82^c^	1,229 ± 59^c^
IL-1β (ng/L)	76.9 ± 4.9 ^a^	96.6 ± 6.4^b^	85.8 ± 5.8^c^	84.1 ± 4.7^c^
IL-6 (pg/L)	275 ± 16^a^	332 ± 15^b^	278 ± 19^a^	280 ± 15^a^
IL-12 (ng/L)	138 ± 7^a^	167 ± 8^b^	159 ± 6^bc^	150 ± 9^c^
IFN-γ (ng/L)	841 ± 76^a^	1485 ± 122^b^	1,206 ± 67^c^	1,103 ± 65^c^
IL-10 (pg/L)	1,437 ± 84^a^	1,014 ± 113^b^	1,257 ± 60^c^	1,366 ± 68^ac^

Values are presented as the mean ± standard deviation (n = 8/group). Normal, mice were treated with saline; Model, mice were treated with 5% DSS; SASP, mice were treated with 50 mg/mL of salicylazosulfapyridine + 5% DSS; LF-HFY06, mice were treated with 1.0 × 10^9^ CFU/mL of LF-HFY06 + 5% DSS; *^a–c^*significant difference (P < 0.05) was indicated by different letters in the same row after Tukey’s test.

### *Rela*, *Nfkbia*, *Tnf*, *Il 1b*, *Il 6*, *Il 10*, and *Ptgs2* mRNA expression levels in colonic tissue

**FIGURE 4:**
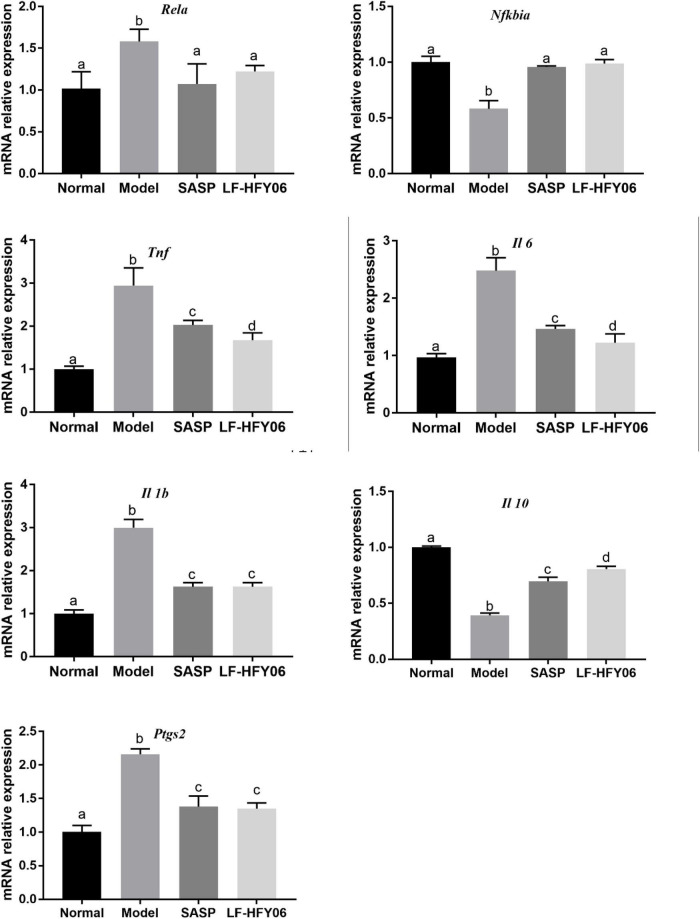
The *Rela, Nfkbia, Tnf, Il 1b, Il 6*, *Il 10*, and *Ptgs2* mRNA expression levels in colon tissues of the different groups. The data are shown as mean ± standard deviation (*n* = 8). ^a–*d*^Significant difference (*P* < 0.05) was indicated by different letters after Tukey’s test.

The NF-κB signaling pathway is closely associated with the occurrence of inflammation. The mRNA expression levels of *Rela*, *Tnf*, *Il 1b*, *Il 6*, and *Ptgs2* were all upregulated in the model group, and the *Nfkbia* and *Il 10* mRNA expression were downregulated in contrast to the normal group (Figure 4, *P* < 0.05). After using LF-HFY06 and SASP to intervene, the *Rela*, *Tnf*, *Il 1b*, *Il 6*, and *Ptgs2* mRNA expression was suppressed, and the mRNA expression of *Nfkbia* and *Il 10* was enhanced as compared to that in the DSS model group (*P* < 0.05). The variation trend revealed that LF-HFY06 may regulate the expression of inflammatory cytokines *via* inhibiting the NF-κB signaling pathway.

## Discussion

DSS is a commonly used reagent for colitis modeling (Strober et al., 2002; Wirtz et al., 2007). DSS induces colonic inflammation in mice, and the resulting colonic congestion, edema, thickening of the colon wall, and the formation of an ulcerated surface will shorten the length of the colon. Therefore, the length of the colon can directly reflect the degree of colitis, which is considered to be negatively correlated with the severity of enteritis (Hu et al., 2021). In the current study, it was found that the length of the colon was shortened by DSS. The histopathological observation indicated that DSS caused the destruction of colonic crypts, as well as injury and apoptosis of colon cells (Hu et al., 2021). From the colon length and pathological examination, the intervention of LF-HFY06 alleviated the shortening of the colon length induced by DSS and assisted in maintaining the structural integrity of the colon. The results are the same as those of studies that have reported that lactic acid bacteria alleviated IBD (Vemuri et al., 2017; Amer et al., 2018).

Excessive activation of oxidative stress is another important mechanism of ulcerative colitis (Piechota-Polanczyk and Fichna, 2014). The enhancement of oxidative stress causes the increase of protein and DNA damage, cell degeneration, necrosis, and then promotes the occurrence and exacerbation of colitis (Zhu and Li, 2012). The TNF, NF-κB, and JNK signaling pathways could be activated by reactive oxygen species (ROS) to induce apoptosis and aggravate intestinal inflammation (Blaser et al., 2016). The body’s antioxidant system consists of enzymatic and non-enzymatic systems (Birben et al., 2012). The enzymatic antioxidant system includes SOD, CAT, and glutathione peroxide. SOD is an antioxidant enzyme that can specifically clear superoxide anions by decomposing it into harmless H_2_O and oxygen molecules (Birben et al., 2012). CAT is an enzyme that catalyzes the decomposition of hydrogen peroxide into oxygen and H_2_O (Birben et al., 2012). MDA is a product of lipid peroxidation, by which nucleic acids and proteins are further damaged. Under the oxidative stress associated with colitis, LF-HFY06 could increase the activities of T-SOD, CAT, and reduce the level of MDA to suppress oxidative stress.

A complex regulatory network of cytokines is involved in colitis (Izcue et al., 2009; Chen and Sundrud, 2016). As a pro-inflammatory cytokine, TNF-α and IL-6 play important roles in the pathogenesis of ulcerative colitis. They induce the release of other cytokines, which aggravate the inflammatory response (Drastich et al., 2011; Billiet et al., 2014; Xiao et al., 2016). IL-1β can promote chemotactic neutrophils and other inflammatory cells to enter the related lesions of the intestine (Mulay et al., 2013). IL-12 is also one of the strongest NK cell-activating factors, and it stimulates NK cells to produce a variety of cytokines, such as IFN-γ, IL-8, and TNF-α (Zelante et al., 2007). Many studies have shown that the IFN-γ level was increased in intestinal inflammation, which exerts immunomodulatory effects (Ferrier et al., 2003). However, IL-10 inhibits inflammation in the microenvironment of the intestinal tissue. It modulates the occurrence and development of intestinal inflammation and maintains the intestinal mucus barrier (Huber et al., 2011; Shouval et al., 2014). DSS stimulates the production of inflammatory factors TNF-α, IL-6, IFN-γ, IL-12, IL-1β, and decreases the levels of anti-inflammatory factor IL-10. LF-HFY06 evidently suppressed the levels of inflammatory factors TNF-α, IL-6, IFN-γ, IL-12, and IL-1β. Meanwhile, it significantly elevated the IL-10 level to inhibit inflammation in DSS-induced colitis.

As a vital transcription factor during the process of the body’s immune response, NF-κB binds to its inhibitory protein IκB in an inactive manner under normal conditions. It is reported that DSS activates NF-κB by accelerating the phosphorylation of IκB protein (Onizawa et al., 2009; Liu et al., 2016). The activated NF-κB further facilitates the release of pro-inflammatory factors, causing severe inflammatory damage to the body (Onizawa et al., 2009; Liu et al., 2016). At the same time, the induced inflammatory cytokines, such as IL-6 and TNF-α, promote the expression of the NF-κB gene to form a positive feedback loop. This triggers an excessive immune response in colon tissues and damages colon mucosa, leading to the occurrence of colitis (Wang et al., 2003; Hu et al., 2014).

The core role of the NF-κB signaling pathway is to regulate the balance between inflammatory factors and anti-inflammatory factors. Studies have reported that *Lactobacillus* sp. relieved colitis symptoms *via* the NF-κB signaling pathway (Chen et al., 2017; Zhou et al., 2019). COX-2 is an inducible enzyme and could catalyze arachidonic acid to produce endogenous prostaglandins, which is coded by *Ptgs2* (Lee et al., 2004). In the current study, the administration of LF-HFY06 suppressed the mRNA expression of *Rela* and elevated the mRNA expression of *Nfkbia* to inhibit the activation of the NF-κB signaling pathway. Then, the mRNA expression of pro-inflammatory cytokines, *Tnf*, *Il 1b*, *Il 6*, and *Ptgs2* was suppressed, and that of anti-inflammatory cytokines *Il 10* was elevated in colon tissue. This indicates that LF-HFY06 may relieve colitis *via* the inhibition of the NF-κB signaling pathway.

According to literature reports, SASP could be decomposed into sulfadiazine and 5-aminosalicylic acid in the effect of intestinal microorganisms. 5-Aminosalicylic acid has antibacterial, anti-inflammatory, and immunosuppressive effects (Zheng, 2006). In this study, LF-HFY06 exerts antioxidant and anti-inflammatory activities by inhibiting the NF-kB signaling pathway. Tables 4, 5 show that insignificant differences were emerged between SASP and LF-HFY06 in regulating the levels of related biochemical indicators. The qPCR analysis showed that higher mRNA expression of the anti-inflammatory IL-10 in the SASP group was observed compared with that of the LF-HFY06 group. However, the expression of the inflammatory gene *Tnf* and *Il 6* in SASP group was higher than those of LF-HFY06. There were no significant differences in other genes. Overall, the ability to regulate gene expression is approximately equal between SASP and LF-HFY06.

In recent years, *Lactobacillus* sp. is used to prevent or alleviate colitis in mice mainly through immunomodulatory effects and regulation of the gut microbiome. *Limosilactobacillus fermentum* CQPC04 could inhibit oxidative stress and inflammation in mice with colitis, and its mechanism of it is to regulate the NF-κB signaling pathway (Zhou et al., 2019). *Limosilactobacillus fermentum* HY01 and *Limosilactobacillus fermentum* IM12 also inhibited the inflammatory symptoms of colitis through this pathway (Chen et al., 2017; Lim et al., 2017). Moreover, it is reported that *Limosilactobacillus fermentum* KBL375 could regulate the immune response and increase the abundance of beneficial microorganisms to change the composition of gut microbiota, thus achieving the purpose of alleviating intestinal inflammatory diseases (Jang et al., 2019). Similarly, Rodríguez-Nogales et al. (2017) reported that *Limosilactobacillus fermentum* CECT5716 could counteract an enrichment in *Bacillus* and *Paenibacillus*, together with a reduction in *Cytophaga*, achieving an anti-inflammatory effect in colitis mice. The regulation of antioxidant levels is also one of the important mechanisms. Chauhan et al. reported that the antioxidant levels were increased by *Limosilactobacillus fermentum* Lf1 to ameliorate colitis (Chauhan et al., 2014). The mechanism of LF-HFY06 in relieving colitis is similar to previous studies. LF-HFY06 ameliorates ulcerative colitis in mice by modulating the NF-κB signaling pathway to downregulate inflammatory cytokines and upregulate anti-inflammatory factors.

Also, short fatty acids (mainly butyric acid) produced by probiotics promote the development of regulatory T cells, which in turn continuously strengthen the mucosal barrier (Schirmer et al., 2019). Second, probiotics can also secrete antibacterial substances, which could inhibit the growth of intestinal pathogens (Schirmer et al., 2019). Those are the directions for further in-depth research on LF-HFY06.

## Conclusion

In our research, 5% DSS was used to establish a colitis model in mice. After intervention with LF-HFY06, the colon tissue damage was significantly alleviated, the antioxidant capacity was enhanced, and the inflammatory response was inhibited. The result of qPCR detection showed that LF-HFY06 may protect against colitis through the NF-κB signaling pathway. The mechanism of LF-HFY06 was involved in the upregulation of *Nfkbia* and *Il 10* mRNA expression, and downregulation of *Rela*, *Tnf*, *Il 1b*, *Il 6*, and *Ptgs2* mRNA expression. However, its experiments on humans have not been carried out yet. In future, the mechanism of LF-HFY06 and relevant basic scientific research will be further analyzed to provide more references for carrying out clinical trials.

## Data availability statement

The original contributions presented in this study are included in the article/supplementary material, further inquiries can be directed to the corresponding author/s.

## Ethics statement

The Animal experiments were approved by the Ethics Committee of Chongqing Collaborative Innovation Center for Functional Food (IACUC Number: 201906002B).

## Author contributions

XZ conceived the study. BL and LY completed the writing of the manuscript, completed the inspection, and modification of the manuscript. BL and YW completed the main experimental work. YW mainly completed data sorting and statistical analysis. All authors discussed the data and reviewed the manuscript.
